# Usability, Ergonomics, and Educational Value of a Novel Telestration Tool for Surgical Coaching: Usability Study

**DOI:** 10.2196/57243

**Published:** 2024-09-10

**Authors:** Parmiss Kiani, Roberta Dolling-Boreham, Mohamed Saif Hameed, Caterina Masino, Andras Fecso, Allan Okrainec, Amin Madani

**Affiliations:** 1 Surgical Artificial Intelligence Research Academy University Health Network Toronto, ON Canada; 2 Temerty Faculty of Medicine University of Toronto Toronto, ON Canada; 3 Department of Surgery University of Toronto Toronto, ON Canada

**Keywords:** augmented reality, AR, surgical training, telestration, tele-stration, surgical training technology, minimally invasive surgery, surgery, surgeon, surgeons, surgical, surgical coaching, surgical teaching, surgical training, telemonitoring, telemonitor, tele-monitoring, tele-monitor, usability, usable, usableness, usefulness, utility, digital health, digital technology, digital intervention, digital interventions

## Abstract

**Background:**

Telementoring studies found technical challenges in achieving accurate and stable annotations during live surgery using commercially available telestration software intraoperatively. To address the gap, a wireless handheld telestration device was developed to facilitate dynamic user interaction with live video streams.

**Objective:**

This study aims to find the perceived usability, ergonomics, and educational value of a first-generation handheld wireless telestration platform.

**Methods:**

A prototype was developed with four core hand-held functions: (1) free-hand annotation, (2) cursor navigation, (3) overlay and manipulation (rotation) of ghost (avatar) instrumentation, and (4) hand-held video feed navigation on a remote monitor. This device uses a proprietary augmented reality platform. Surgeons and trainees were invited to test the core functions of the platform by performing standardized tasks. Usability and ergonomics were evaluated with a validated system usability scale and a 5-point Likert scale survey, which also evaluated the perceived educational value of the device.

**Results:**

In total, 10 people (9 surgeons and 1 senior resident; 5 male and 5 female) participated. Participants strongly agreed or agreed (SA/A) that it was easy to perform annotations (SA/A 9, 90% and neutral 0, 0%), video feed navigation (SA/A 8, 80% and neutral 1, 10%), and manipulation of ghost (avatar) instruments on the monitor (SA/A 6, 60% and neutral 3, 30%). Regarding ergonomics, 40% (4) of participants agreed or strongly agreed (neutral 4, 40%) that the device was physically comfortable to use and hold. These results are consistent with open-ended comments on the device’s size and weight. The average system usability scale was 70 (SD 12.5; median 75, IQR 63-84) indicating an above average usability score. Participants responded favorably to the device’s perceived educational value, particularly for postoperative coaching (agree 6, 60%, strongly agree 4, 40%).

**Conclusions:**

This study presents the preliminary usability results of a novel first-generation telestration tool customized for use in surgical coaching. Favorable usability and perceived educational value were reported. Future iterations of the device should focus on incorporating user feedback and additional studies should be conducted to evaluate its effectiveness for improving surgical education. Ultimately, such tools can be incorporated into pedagogical models of surgical coaching to optimize feedback and training.

## Introduction

Telementoring studies found technical challenges in achieving accurate and stable annotations using commercially available telestration software intraoperatively [1-3].

The first challenge is the dynamic nature of the video feed; there are frequent laparoscopic camera movements, field of view changes, and deformation of anatomic structures due to the mobilization and retraction of anatomical structures, and maneuvering of the camera [4,5].

Mitigation strategies during coaching activities included freezing the video and converting it to still images [6]. This is not practical for real-time intraoperative coaching by surgeons and greatly increases the time spent on the activity to stop and restart the session during annotation.

Previous usability studies that used telestration [6-8] used a trackpad, mouse, or touchscreen during annotation mode and found that the trackpad or mouse performed best in the delineation of structures, while the touch screen was superior in conveying directional information [7]. Further, 1 study compared the usability of similar telestration devices with conventional interfacing devices such as a computer and mouse, and a tablet and stylus [4]. With the advancement of technology in the gaming world, new virtual reality (VR) or augmented reality systems have emerged as viable solutions for the development of systems for telestration.

To address the educational gap in teaching minimally invasive procedures, a wireless handheld telestration device was developed to facilitate dynamic user interaction with live video streams. This study examines the usability of a first-generation handheld wireless telestration platform.

Continuing professional education activities such as surgical coaching provide opportunities for the continued acquisition of new techniques and professional expertise.

Telestration is a technique for teaching whereby instructors annotate images or videos to enhance the learning experience for surgical trainees [9]. This technique has shown promise for improving surgical skills more effectively than traditional verbal coaching across a broad range of metrics including faster task completion, reduced coaching time, better surgical performance, and greater trainee confidence [7,10-12]. Previous telestration studies have highlighted the importance of mentor-mentee communication in surgical training [7,11,13].

In the setting of laparoscopic surgery, the learning curve for trainees tends to be greater as the surgical field cannot be directly visualized or palpated as in open surgeries and directly pointing at surgical display screens for teaching and coaching activity raises concerns of sterility. Instead, feedback and guidance are typically verbally described without the ability to make direct references, which may lead to greater trainee confusion, miscommunication, and ultimately reduced efficacy of the teaching process. This is especially relevant with the shift to minimally invasive surgeries, which provide numerous benefits to patients over open surgeries [14]. Given the benefits of telestration seen for trainees and the adoption of minimally invasive surgery for increasingly more complex procedures, it is critical to develop better tools to improve the acquisition of these skills.

To augment the coaching experience, a wireless handheld telestration tool was developed to better address dynamic on-demand teaching requirements both intraoperatively during a procedure and postoperatively on a recorded surgical video. This study presents the usability results of a first-generation handheld wireless telestration platform.

## Methods

### Hardware Design

A wireless handheld telestration device prototype, hereon referred to as the pen, was designed and manufactured to enable the user (ie, surgeon coach) to interact with the surgical display field during coaching activities in both intraoperative and postoperative settings. This prototype uses a proprietary augmented reality platform.

**Figure 1:**
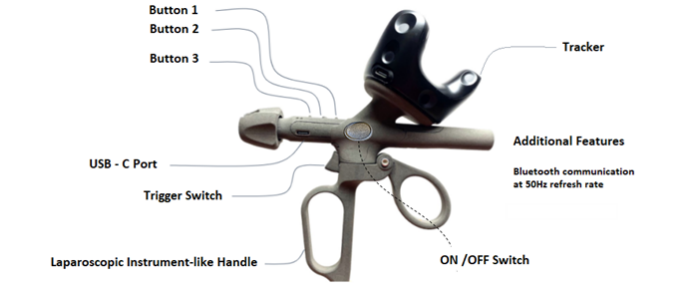
Telestration device.

The pen (Figure 1) consists of two main parts: (1) a motion tracker mounted to (2) a 3D printed controller. The motion tracker and the controller have separate charging systems and on/off buttons. The controller has 3 buttons with various functions, a trigger, and a Maryland grasper handle to create a more realistic user experience. The pen weighs 152.76 g.

### Software Design

The four core functions of this video-based coaching platform are (1) free hand annotation, (2) cursor navigation, (3) overlay and manipulation of ghost (avatar) instruments, and (4) hand-held video feed navigation.

**Figure 2:**
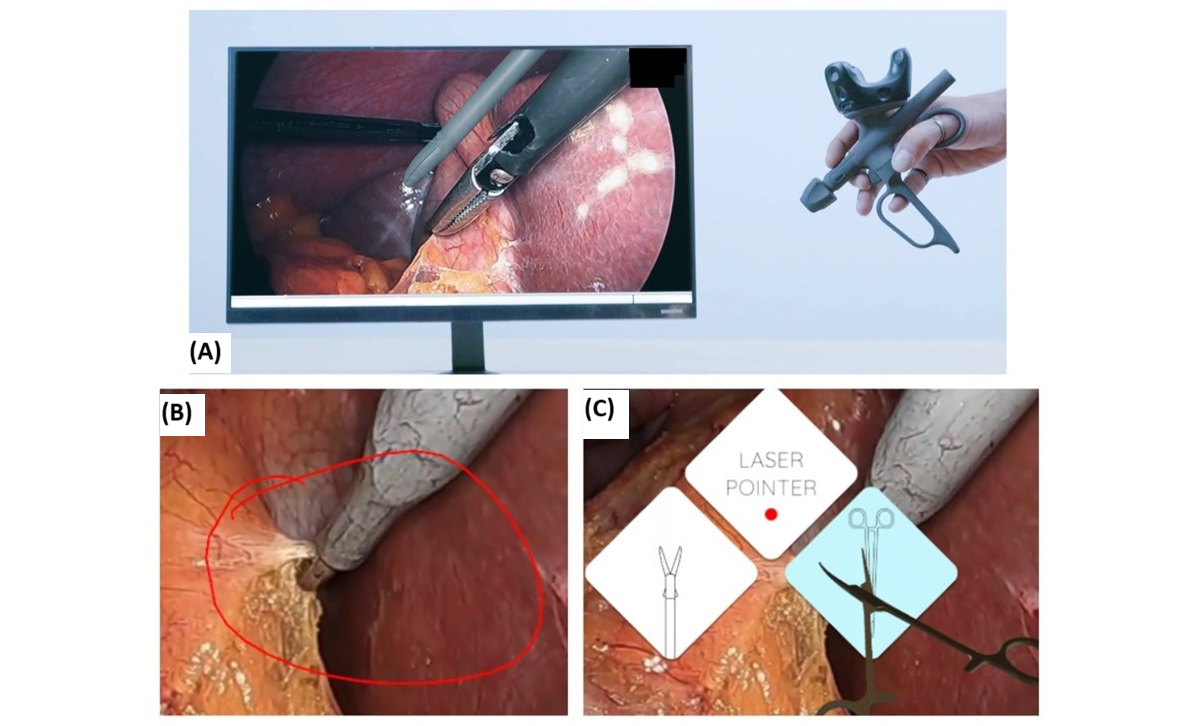
(A) Telestration device used for video-based coaching, (B) annotation feature demonstration, and (C) telestration software menu options.

These functions may be completed on a live or recorded video feed. To achieve these core functions, the telestration software menu (Figure 2C) allows the user to choose from 3 interactive tools: a laser pointer for cursor navigation and annotation (Figure 2B), and 2 digital avatar instruments for dynamic coaching and positioning instructions: 1 for laparoscopy (designed to resemble a Maryland Grasper) and 1 for open surgery (designed to resemble a needle driver; Figure 2).

### System Platform Integration

The telestration platform integrates a legacy guidance and tracking system using: Vive (HTC Corp) and SteamVR (Valve Corp).

**Figure 3:**
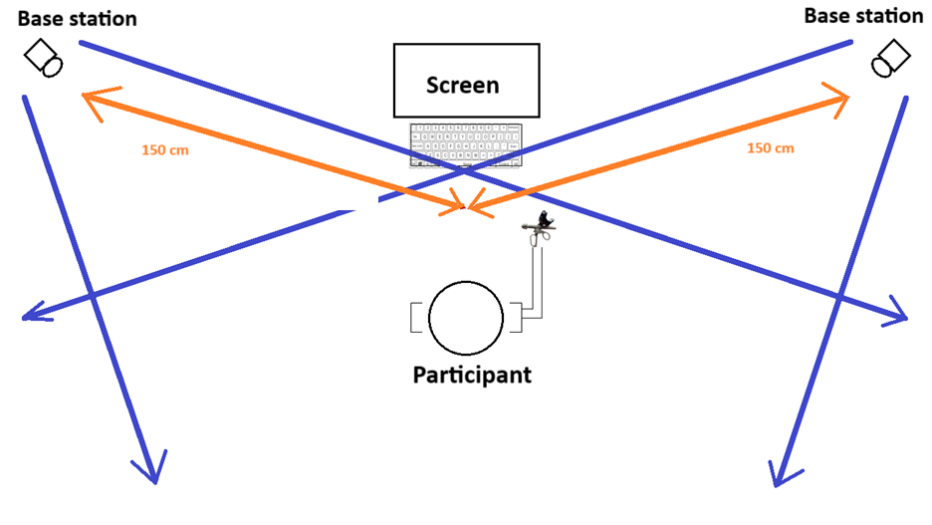
Usability study room set-up.

The tracker unit is mounted onto the telestration pen’s hardware (Figure 1). The tracker has multiple diodes that detect the infrared signals emitted by the SteamVR lighthouse units. Lighthouse units are externally powered devices mounted at locations of high visibility around the physical space to detect and track the telestration device’s movements by the user. The number of lighthouse units is determined by the size of the room where the telestration pen is implemented. A total of 2 lighthouse units were placed 1.5 m apart to achieve optimal performance as per the manufacturer’s instructions (Figure 3). The SteamVR system appears to designate 1 lighthouse as the primary source of data and another as a secondary source [15]. Figure 3 illustrates the room setup.

The orientation of the lighthouses per the object being tracked is crucial to the accuracy of the system. The tracking accuracy is greatest with the pen positioned orthogonally to it. The maximum distance between the lighthouse and the tracker is the maximum distance to work effectively which is 7 m [15].

Therefore, for this study, a total of 2 lighthouse units were placed 1.5 m apart to achieve optimal performance as per the manufacturer’s instructions (Figure 3).

The lighthouse units (“base stations”) emit infrared light which is detected by diodes present on the tracker and this information is converted to positional data by SteamVR software.

### Usability Study Design

The International Organization of Standardization defines usability as “the extent to which a system, product or service can be used by specified users to achieve specified goals with effectiveness, efficiency and satisfaction in a specified context of use” [16]. Testing a device with end users is essential for a comprehensive evaluation of its usability. Therefore, the usability study was conducted in 3 phases (presurvey, usability testing, and post survey) and included an informal debrief at the end of the session.

### Participants

All physicians who play a role in surgical education were allowed to participate. Participants were surgeons and surgical trainees recruited from a multi-cite academic teaching hospital in Toronto, Ontario, Canada.

### Data Collection

#### Phase 1 (Presurvey)

A short questionnaire was administered to gather baseline information regarding demographics and prior experience with VR and telestration technologies.

#### Phase 2 (Usability Testing)

The functional capability of the telestration device was designed by surgeon educators based on the needs they noted in real-world coaching situations. Scenarios were then prepared to evaluate the 4 core functions of the device in those situations. A study facilitator guided participants through the scenarios in a representative simulated environment. Examples of tasks include starting the device, menu selection, tool navigation and overlay, annotating and drawing, moving video playback or pause, and so on. A complete list of tasks is outlined in Textbox 1.

**Tasks for telestration function assessment.**

**Standard tasks**

- App launch and device calibration: participants were asked to start a preselected video displaying certain clips from a laparoscopic cholecystectomy.
- Play or pause video feed: participants were asked to play and pause the video at certain points throughout the session.

**Video feed navigation**

- Fast forward and rewind: participants were asked to fast forward and rewind the video to predetermined points of the video throughout the session.

**Overlay and manipulation of ghost avatar instrument**

- Menu-instrument selection: participants were asked to open the menu and select either the laser pointer or the grasper multiple times throughout the session.
- Rotate grasper tip: participants were asked to rotate the grasper tip.
- Open and close grasper tip: participants were asked to open and close the grasper tip.

**Freehand annotation**

- Annotation: participants were asked to open and close the grasper tip.
- Erase annotation: participants were asked to annotate where they would perform a dissection at a specific moment of a procedure and to circle the cystic duct.

#### Phase 3 (Postsurvey)

Participants completed the system usability scale (SUS) after testing was completed [17]. This scoring system compiles the responses from a series of 10 questions that cover topics including device complexity, ease of use, the learning curve required, and user confidence [18]. In addition to this, a questionnaire was developed by the researchers to gather the attitudes and opinions of the participants on usability, satisfaction, ergonomics, ease of task completion, confidence, and the perceived educational value of the device, using a 5-point Likert scale.

### Procedure

Before the start, this study’s room was set up as shown in Figure 3, the VIVE tracker was calibrated to the room using SteamVR. A member of the research team was this study’s facilitator. Participants began the session by completing the presurvey (phase 1). Next, the participants were asked to hold a device such as a laparoscopic grasper. The facilitator trained the participants on the functionality of the device and provided the participants with a quick tip sheet to reference during the usability test.

Participants were asked to select a video of a laparoscopic cholecystectomy that was provided for this study’s purpose. Participants were then asked to calibrate the controller by following the instructions on the screen. Participants were first asked to use the telestration device while standing to emulate its use in an operating room, followed by a seated position for an office or boardroom setting. The facilitator guided the participant to complete tasks in Textbox 1, testing the 4 core functions of the device in a standing position and ending the video on completion. Upon study completion, participants completed phase 3 of this study.

### Outcomes

Outcomes measured in this study were perceived usability, ergonomics, overall satisfaction, and the perceived educational value of the telestration device.

### Data Analysis

Descriptive statistics were used, and qualitative variables were reported as frequencies and percentages.

The results of the SUS were analyzed according to the scoring procedure documented by Brooke [17]. A product with a SUS score greater than 70 is considered to have above-average usability [18].

### Ethical Considerations

Informed consent was taken before the start of this study. This study was approved by the University Health Network’s (UHN) Research Ethics Board (22-5556; education research protocol dated September 14, 2022).

## Results

### Phase 1 Demographics

A total of 9 surgeons and 1 senior resident (n=10) participated (5 males and 5 females). The average age of participants was 36.4 (SD 6) years with a mean of 7 (SD 6.41) years of practice. All participants reported being right-handed. The majority of participants (7 out of 10, 70%) reported no previous telestration system experience. Only 1 participant reported having received training with a surgical VR system before this study. Most participants (5 out of 10, 50%) reported not using VR and gaming consoles in the last 12 months (3 out of 10, 30% monthly and 2 out of 10, 20% rarely).

### About SUS

This study’s average SUS was 70 (SD 12.5) with a median of 75 (IQR 63-84).

### Overall Satisfaction

Participants were asked to rate their overall satisfaction using a 5-point Likert scale where 10% (1 out of 10) of participants were completely satisfied, 50% (5 out of 10) were very satisfied, 20% (2 out of 10) were moderately satisfied, and 20% (2 out of 10) slightly satisfied.

### Ergonomics

When asked about the intuitiveness of the device, most participants strongly agreed or agreed (SA/A) that the device was intuitive (5 out of 10, 50%), 40% (4 out of 10) felt neutral, and 10% (1 out of 10) disagreed or strongly disagreed (D/SD). Ergonomics was further assessed by asking participants to respond specifically regarding the pen’s physical comfort (4 out of 10, 40% SA/A; 4 out of 10, 40% neutral; and 2 out of 10, 20% D/SD), the weight of the pen (6 out of 10, 60% SA/A and 4 out of 10, 40% D/SD), and the ability to use the physical features (buttons and trigger; 4 out of 10, 40% SA/A; 3 out of 10, 30% neutral; and 3 out of 10, 30% D/SD).

Participants were also asked if they preferred completing this study’s tasks while in a seated or standing position. In total, 4 participants preferred to be seated, 3 preferred to be standing, and 3 had no preference at all. When asked about their confidence in completing the tasks correctly, 90% (9 out of 10) and 80% (8 out of 10) SA/A that they correctly completed the tasks seated and standing, respectively.

### Open Ended Survey Responses

Participants were asked to discuss features of the pen and telestration system that they felt were design strengths as well as areas of improvement (Textbox 2). Of the 8 participants who responded, 3 participants reported the ability to annotate on the screen as a good feature of the device. Regarding areas for improvement, 6 participants indicated to have the location of the buttons moved.

**Open feedback responses from the participants regarding the virtual reality system.**

**What 3 things are good about the virtual reality system?**

- Hands-on teaching, not invasive, and may be widely used.
- It allows you to draw on the image on the screen, it allows you to demonstrate the orientation of instruments, and it allows you to fast forward and rewind.
- Felt accurate in terms of location of the pointer, intuitive to use, and realistic feeling.
- Great response time, useful for annotation, and innovative.
- Teaching.
- Easy to use.
- Ability to annotate, erase, and select instruments.
- Relatively easy to use after a short guidance, it is cool, innovative, and less stressful.

**What 3 things can be improved in the virtual reality system?**

- Button placement, precision, and ergonomics.
- The actual instrument itself (the weight of it and location of buttons), the directionality of fast forward or rewind (to make it more intuitive), and the function of the hand holds.
- Location of buttons.
- Calibration of pen not in line with pen, instrument heavy, and hard to hold for small hands to reach the top buttons.
- Fulcrum on table.
- Calibration, weight, and function of finger loops.
- Ergonomics (handle is not needed), buttons are not easy to use, and tracking sensor drift.
- The [pen] is a little uncomfortable, the pen is heavy, and when sitting it is hard to see the animation for the Maryland.

### Postsurvey Satisfaction Responses

Participants were asked to rate their level of agreement with statements on the standard tasks performed in this study (Table 1).

The majority of participants (6 out of 10, 60%) found the setup tasks (launching the video and device calibration) easy to complete, while 30% (3 out of 10) of participants found it difficult and 10% (1 out of 10) felt neutral. On the other hand, 90% (9 out of 10) of participants agreed that fast-forwarding and rewinding, as well as annotating, were easy, and 100% (10 out of 10) of participants agreed that erasing annotations was easy.

**Table 1:** Ease of use in completing tasks testing the device’s various functions.

Function		Agree or strongly agree, n (%)	Neutral, n (%)	Disagree or strongly disagree, n (%)
**Standard tasks**				
	Difficulty completing initial setup: launch and calibration	3 (30)	1 (10)	6 (60)
	Play or pause the video feed	8 (80)	2 (20)	0 (0)
**Video feed navigation**				
	Fast forward and rewind	9 (90)	1 (10)	0 (0)
**Overlay and manipulation of ghost instrument**				
	Menu-instrument selection	7 (70)	3 (30)	0 (0)
	Rotate grasper tip	4 (40)	5 (50)	1 (10)
	Open and close the grasper tip	7 (70)	2 (20)	1 (10)
**Freehand annotation**				
	Annotation	9 (90)	0 (0)	1 (10)
	Erase annotation	10 (100)	0 (0)	0 (0)

### Confidence Levels (Pre- Versus Poststudy Comparison)

#### Overview

Participants were asked to rate their confidence in the system features at baseline and post study using a scale of 1 to 5, where 1 is not confident and 5 is very confident.

#### Set Up and Training

Participants were also asked to rate their level of confidence in their technical ability to independently set up a VR or gaming system in the post study. The majority of participants (6 out of 10, 60%) rated their confidence in system setup at a 4 out of 5, while 20% (2 out of 10) rated it a 5 out of 5, and another 20% (2 out of 10) rated it a 3 out of 5. In addition, the majority of participants felt that the system training completed by the facilitator was adequate (8 out of 10, 80%), with 20% (2 out of 10) of participants feeling the training period was too short. Lastly, the majority rated the quality of the training provided for study purposes as excellent 1 out of 10, 10%; very good 6 out of 10, 60%; and good 3 out of 10, 30%.

#### Navigation

The majority of participants reported a confidence rating of 4 at baseline for navigating accurately and realistically (confidence of 5: 1 out of 10, 10%; confidence of 4: 8 out of 10, 80%; confidence of 3: 1 out of 10, 10%), which then increased to a rating of 5 post study (confidence of 5: 5 out of 10, 50%; confidence of 4: 4 out of 10, 40%; and confidence of 3: 1 out of 10, 10%). No participant reported a confidence of 2 or less in either the pre or post study.

#### Instrument Overlay

The greatest positive change (40% increase) between pre and post study in confidence rating was present for the overlay of the digital tool. Confidence ratings for this category prestudy were (confidence of 5: 1 out of 10, 10%; confidence of 4: 4 out of 10, 40%; confidence of 3: 4 out of 10, 40%; confidence of 2: 0 out of 10, 0%; and confidence of 1: 1 out of 10, 10%), while post study were (confidence of 5: 1 out of 10, 10%; confidence of 4: 8 out of 10, 80%; confidence of 3: 1 out of 10, 10%; confidence of 2: 0 out of 10, 0%; and confidence of 1: 0 out of 10, 0%).

#### Annotations

The confidence levels for performing annotations were highly rated for both pre- (confidence of 5: 2 out of 10, 20%; confidence of 4: 7 out of 10, 70%; and confidence of 3: 1 out of 10, 10%) and post study (confidence of 5: 4 out of 10, 40% and confidence of 4: 6 out of 10, 60%).

#### Video Feedback

Regarding video feed playback or pause reviewing functions, confidence levels remained unchanged pre to post study (confidence of 5: 3 out of 10, 30%; confidence of 4: 6 out of 10, 60%; and confidence of 3: 1 out of 10, 10%).

#### Select and Change Tools

Lastly, while no participant felt very confident selecting or changing tools in the prestudy (confidence of 5: 0 out of 10, 0%; confidence of 4: 7 out of 10, 70%; and confidence of 3: 3 out of 10, 30%), 30% (3 out of 10) of participants rated that they felt very confident to select or change tools in the post study phase (confidence of 5: 3 out of 10, 30%; confidence of 4: 5 out of 10, 50%; and confidence of 3: 2 out of 10, 20%). When participants were also asked for their level of agreement about the ease of switching between digital tools, 70% (7 out of 10) of participants SA/A that it was easy to do and 30% (3 out of 10) of participants felt neutral about it.

### Educational Value

**Figure 4:**
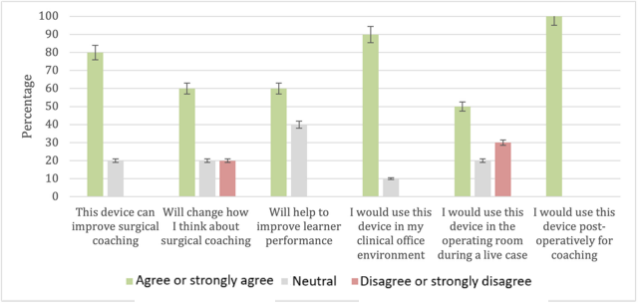
Participant agreement responses on perceived educational value.

Participants were asked to rate the perceived educational value of the device using a 5-point Likert agreement scale. All participants agreed that they would use this device for postoperative coaching, while only 50% (5 out of 10) of participants agreed that they would use the device in an intraoperative setting. Additional questions were asked regarding the use for educational purposes described in Figure 4.

### Technical Difficulties

Lastly, participants were asked to report on technical difficulties experienced while participating in this study. While 40% (4 out of 10) of participants reported experiencing a delay or lag with the device positioning, only 10% (1 out of 10) of participants reported experiencing difficulties loading this study’s video and 10% (1 out of 10) also noticed instructional text not displaying correctly. When participants were asked about the device’s ability to track their hand movements accurately, 60% (6 out of 10) SA/A that it did and 40% (4 out of 10) of participants felt neutral about it.

## Discussion

### Principal Findings

A novel telestration device for surgical coaching was designed to enhance the surgeon coach and learner experience in the context of laparoscopic surgeries. This device enables a dynamic interaction with surgical display monitors with a free-hand annotation function and live overlay of 3D digital laparoscopic tool avatars.

Previously described devices and systems for telementoring had usability challenges that this device aims to mitigate. This study aimed to evaluate the overall satisfaction and usability of the first-generation prototype telestration device for use in surgical coaching activities. In terms of demographics, our study had an equal distribution of women and men, which strengthens the validity of our results.

The use of evaluation tools such as SUS during the development and testing process of user interface apps is commonly recommended in the literature [7,10]. The SUS reported an average score of 70 (SD 12.5) with a median score of 75 (IQR 63-84) indicating an above-average usability rating in comparison to thousands of other devices and systems [18]. Additionally, 60% (6 out of 10) of participants were either completely or very satisfied with the device overall. This is an encouraging result of our first iteration prototype.

On the other hand, those who rated their overall satisfaction less than this, commented on the device’s ergonomics, including the button placement, finger loops, and weight, as well as the device’s precision and lagging experience. These comments on ergonomics are likely why participants had lower confidence in the post study to select or change tools compared to the other tasks. Additionally, while most users agreed that the device’s weight was comfortable, only 40% (4 out of 10) felt the device’s shape was physically comfortable to hold. Furthermore, 40% (4 out of 10) felt that the buttons were hard to reach; a theme that also emerged in the open feedback responses where 1 user commented “instrument … hard to hold for small hands to reach the top buttons.” Comments were also made about the usefulness of the handle piece. While confidence levels of completing study tasks accurately were similar in either position, participants did prefer using the device while sitting. The ergonomic feedback is in line with the SUS scores reported. With ergonomics being a priority for surgeons, future iterations of this device will aim at improving these scores.

With regards to task completion, including video stream controls (play or pause), annotation, and tool avatar manipulation, was generally very positive; the majority of them were considered “easy” to complete. All tasks were completed successfully with the provided training even though participants had never interacted with the device before this study. In addition, many of the participants did not experience any technical difficulties performing all the tasks—no major bugs were identified in this study. Only 40% of participants reported experiencing a lag during the usability testing period, and the time required to complete a task was comparable to that of this study’s facilitator (trainer). Therefore, in future iterations of the device and software, addressing the lag experienced by users is of importance.

With regards to task completion, including video stream controls (play or pause), annotation, and tool avatar manipulation, was generally very positive; the majority of them were considered “easy” to complete. All tasks were completed successfully with the provided training even though participants had never interacted with the device before this study. In addition, the majority of the participants did not experience any technical difficulties performing all of the tasks listed in Textbox 1, as only 40% (4 out of 10) participants reported experiencing a lag during the usability testing period, and the time required to complete a task was comparable to that of this study’s facilitator (trainer). Therefore, in future iterations of the device and software, addressing the lag experienced by users is of importance.

Furthermore, participants found the majority of tasks easy to complete, most notably video manipulation (pause or play at 8 out of 10, 80% and fast-forward or rewind at 9 out of 10, 90%) and annotation (draw at 9 out of 10, 90% and erase at 10 out of 10, 100%). Overall, about task completion, this device demonstrates an acceptable level of usability; all tasks were completed successfully, and the majority of them were considered easy to complete.

In analyzing the open feedback responses, participants used language including “easy to use,” “great response time,” “accurate,” “intuitive,” and “realistic” to describe the device when allowed to provide open and anonymous feedback. This positive feedback is highly encouraging and highlights important themes relevant to usability including an acceptable level of complexity and realistic experience.

Lastly, participants evaluated the perceived educational value of the device with an overwhelming majority (8 out of 10, 80%) of users agreeing that this device can improve surgical coaching, especially in the postoperative setting (10 out of 10, 100%). However, only 50% (5 out of 10) of participants agreed that they would use the device live in an operating room, the main setting in which we intended this device to be used. Thus, participant hesitancy is an important goal of future usability assessments of the telestration device and perhaps would be better understood with testing in an operating room.

### Study Limitations

While a sample size of 4 to 5 participants in usability studies is usually adequate in detecting 80% of system issues, a limitation of our study was its smaller sample size [19]. Another limitation of our study was that the participants were asked about their thoughts on the usability of the device in different settings, specifically intraoperatively. As this study was conducted within an office setting and not within the operating room, it limits the applicability of the answers to being a preliminary thought rather than an actual observation in the asked-about scenario.

### Future Considerations

Overall, the results of this first iteration study indicate our novel telestration device has a strong degree of usability, general user satisfaction, and potential concerning surgical skills coaching. Therefore, we have determined the prototype to have met a satisfactory threshold to merit further development and refinement.

Further improvements will focus on ergonomics with effort dedicated to making the device lighter and relocating the buttons to a more accessible location. Based on participant input, future iterations should also investigate either adding functionality to the device’s handle or potentially removing it altogether. This would address the majority of constructive criticisms from users. From the software perspective, improvements of priority include refinements in the software to allow for a more simplified app launch and calibration as this was the main task of difficulty for our participants.

Future studies should evaluate the educational value of the device in the operating room setting and further evaluate its effectiveness in enhancing the surgeon coach and trainee experience.

### Conclusion

In conclusion, preliminary usability testing of a prototype telestration device for surgical coaching has demonstrated above-average usability and positive feedback regarding the perceived educational value and task completion. Future improvements should focus on ergonomics and design, namely weight and button location, as well as app launch and calibration. The next steps following usability testing can include the assessment of the educational value of the telestration device.

## Abbreviations

D/SD: disagreed or strongly disagreed
SA/A: strongly agreed or agreed
SUS: system usability scale
UHN: University Health Network
VR: virtual reality
